# Shutdown of HIV-1 Transcription in T Cells by Nullbasic, a Mutant Tat Protein

**DOI:** 10.1128/mBio.00518-16

**Published:** 2016-07-05

**Authors:** Hongping Jin, Dongsheng Li, Haran Sivakumaran, Mary Lor, Lina Rustanti, Nicole Cloonan, Shivangi Wani, David Harrich

**Affiliations:** a Department of Cell and Molecular Biology, QIMR Berghofer Medical Research Institute, Herston, Queensland, Australia; b Department of Genetics and Computational Biology, QIMR Berghofer Medical Research Institute, Herston, Queensland, Australia

## Abstract

Nullbasic is a derivative of the HIV-1 transactivator of transcription (Tat) protein that strongly inhibits HIV-1 replication in lymphocytes. Here we show that lentiviral vectors that constitutively express a Nullbasic-ZsGreen1 (NB-ZSG1) fusion protein by the eEF1α promoter led to robust long-term inhibition of HIV-1 replication in Jurkat cells. Although Jurkat-NB-ZSG1 cells were infected by HIV-1, no virus production could be detected and addition of phorbol ester 12-myristate 13-acetate (PMA) and JQ1 had no effect, while suberanilohydroxamic acid (SAHA) modestly stimulated virus production but at levels 300-fold lower than those seen in HIV-1-infected Jurkat-ZSG1 cells. Virus replication was not recovered by coculture of HIV-1-infected Jurkat-NB-ZSG1 cells with uninfected Jurkat cells. Latently infected Jurkat latent 6.3 and ACH2 cells treated with latency-reversing agents produced measurable viral capsid (CA), but little or none was made when they expressed NB-ZSG1. When Jurkat cells chronically infected with HIV-1 were transduced with lentiviral virus-like particles conveying NB-ZSG1, a >3-log reduction in CA production was observed. Addition of PMA increased virus CA production but at levels 500-fold lower than those seen in nontransduced Jurkat cells. Transcriptome sequencing analysis confirmed that HIV-1 mRNA was strongly inhibited by NB-ZSG1 but indicated that full-length viral mRNA was made. Analysis of HIV-1-infected Jurkat cells expressing NB-ZSG1 by chromatin immunoprecipitation assays indicated that recruitment of RNA polymerase II (RNAPII) and histone 3 lysine 9 acetylation were inhibited. The reduction of HIV-1 promoter-associated RNAPII and epigenetic changes in viral nucleosomes indicate that Nullbasic can inhibit HIV-1 replication by enforcing viral silencing in cells.

## IMPORTANCE

HIV-1 infection is effectively controlled by antiviral therapy that inhibits virus replication and reduces measurable viral loads in patients below detectable levels. However, therapy interruption leads to viral rebound due to latently infected cells that serve as a source of continued viral infection. Interest in strategies leading to a functional cure of HIV infection by permanent viral suppression, which may be achievable, is growing. Here we show that a mutant form of the HIV-1 Tat protein, referred to as Nullbasic, can inhibit HIV-1 transcription in infected Jurkat T cell to undetectable levels. Analysis shows that Nullbasic alters the epigenetic state of the HIV-1 long terminal repeat promoter, inhibiting its association with RNA polymerase II. This study indicates that key cellular proteins and pathways targeted here can silence HIV-1 transcription. Further elucidation could lead to functional-cure strategies by suppression of HIV transcription, which may be achievable by a pharmacological method.

## INTRODUCTION

Although HIV-1 infection can be controlled by antiretroviral drugs, there is still no cure for HIV-1-infected patients. This is due to the large numbers of latently infected cells that can continue to spread infectious virus when antiretroviral therapies are suspended. Latently infected cells in patients may not express HIV-1 at all or express it at very low levels, but virus production can be increased by activation of the HIV-1 long terminal repeat (LTR) promoter by mechanisms not completely defined. Understanding the breadth of the molecular mechanisms that regulate HIV-1 latency is urgently needed in order to develop novel strategies to either eliminate or control HIV-1 cellular reservoirs (1).

Following infection of susceptible human cells, HIV-1 converts the viral genomic RNA into a double-stranded DNA copy that, following integration into the chromosome, is transcribed by RNA polymerase II (RNAPII) just like other cellular genes (2). Robust transcription of the integrated proviral DNA by RNAPII is regulated at the step of transcriptional elongation by the positive transcription elongation factor P-TEFb, a cyclin-dependent kinase complex composed of cyclin T1 and Cdk9 (3). In the absence of P-TEFb, HIV-1 transcription yields short viral RNAs typically less than 100 nucleotides long. HIV-1 Tat directs high levels of full-length viral mRNA transcription in cells by recruiting free P-TEFb, as well as liberating it from the 7SK snRNP, which enables binding of P-TEFb to the transactivation response (TAR) element in short nascent HIV-1 mRNA (4). From here, Cdk9 phosphorylates the RNAPII carboxy-terminal domain, the 5,6-dichloro-1-β-d-ribofuranosylbenzimidazole sensitivity-inducing factor, and negative elongation factor, and these events are critical for a transition to productive synthesis of full-length viral mRNAs and leads to virus replication (5). However, HIV-1 transcription can be suppressed in a manner that establishes latent but replication-competent provirus in resting memory CD4^+^ T cells by mechanisms not yet fully defined.

HIV-1 latency involves complex interactions of cellular and viral factors. Some recent evidence suggests that latency establishment may be a transcription factor restriction phenomenon operating directly on the viral promoter (6), on chromatin organization via histone and DNA modification (7, 8), and in opposition to Tat-TAR RNA transactivation (1). Posttranscriptional mechanisms of HIV-1 latency involving splicing and RNA transport have also been implicated (9–14). While the activation status of a cell (cell state) can influence latency by multiple molecular mechanisms that generally suppress Tat positive feedback (15, 16), establishment of latency in activated cells is possible (17, 18).

Tat plays an important role in the regulation of HIV-1 latency and especially in the reactivation of HIV-1 from resting T cells. A recent study investigated HIV-1 latency where cell state activation and productive HIV-1 gene expression were decoupled (18). According to that study, “viral circuitry” autonomously regulates viral latency in conjunction with stochastic fluctuations in gene expression. That study supports a model in which establishment of HIV-1 latency is independent of the cell state and Tat feedback alone is sufficient to rescue HIV-1 transcription in cells. This requires the cellular Tat interactors P-TEFb and the super elongation complex (SEC, which includes P-TEFb as a subunit) to promote the elongation of HIV-1 mRNA by RNAPII as previously described (19). Given the central importance of Tat in the regulation of latency, inhibitory or stimulatory molecules that act on the Tat–P-TEFb interactions should be capable of preventing or stimulating HIV-1 activation in T cells regardless of the cell state.

Previously, we described a transdominant negative mutant Tat protein named Nullbasic (20). Nullbasic is a two-exon form of Tat that is 101 amino acids long and has a substitution in the Tat arginine-rich motif (ARM) composed primarily of glycine. Nullbasic can inhibit replication of HIV-1 when expressed in human cells (21). This involves three independent mechanisms. First, Nullbasic is packaged into HIV-1 particles, where it can bind reverse transcriptase and inhibit reverse transcription (RT) in newly infected cells (22). Second, Nullbasic binds to the RNA helicase DDX1 in the nuclei of cells and thereby inhibits Rev (23). Third, Nullbasic can inhibit transactivation by competing with wild-type Tat for interaction with P-TEFb (20, 24).

In this study, we demonstrate that Nullbasic can durably protect Jurkat cells from *de novo* HIV-1 infection by blocking virus replication; it could also strongly suppress HIV-1 replication in productively infected Jurkat cells by inhibiting HIV-1 transcription, and it can prevent reactivation of HIV-1 in latently infected Jurkat and ACH2 cells. Our results suggest that stable expression of Nullbasic can break the “viral circuitry” required for active HIV-1 transcription.

## RESULTS

### NB-ZSG1 prevents HIV-1 replication in Jurkat cells.

To achieve stable expression, a Nullbasic-ZsGreen1 (NB-ZSG1) fusion protein or ZsGreen1 (ZSG1) alone was inserted into the lentiviral vector pSicoR-EF1a (25), which expressed the inserted genes by using the constitutively active EF-1α promoter. NB-ZSG1 virus-like particles (VLPs) were produced by cotransfection of HEK293T cells with individual pSicoR vectors along with pCMVΔ8.91 and pCMV-VSV-G (see Fig. S1A in the supplemental material). Jurkat-NB-ZSG1 and Jurkat-ZSG1 cells were obtained by transduction of Jurkat cells with NB-ZSG1 and ZSG1 VLPs and isolation by fluorescence-activated cell sorting (FACS). Cell purity was analyzed for NB-ZSG1 or ZSG1 expression by flow cytometry (see Fig. S1B). Expression of NB-ZSG1 was confirmed by Western blotting with an anti-Tat antibody (see Fig. S1C). No significant effect of NB-ZSG1 or ZSG1 expression on cell proliferation and viability was observed in a 72-h 3-(4,5-dimethylthiazol-2-yl)-5-(3-carboxymethoxyphenyl)-2-(4-sulfophenyl)-2*H*-tetrazolium (MTS)-based assay (see Fig. S1D).

**FIG 1:**
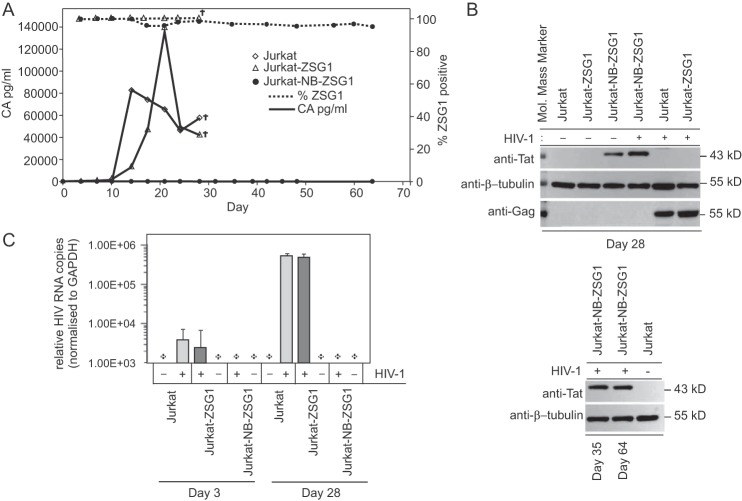
HIV-1 replication is not detected in Jurkat-NB-ZSG1 cells. (A) The Jurkat-NB-ZSG1 and Jurkat-ZSG1 stable cell lines or parental Jurkat cells were infected with HIV-1, and the infected cells were cultured for up to 64 days. Supernatant was sampled as indicated, and the concentration of CA was measured by ELISA with a threshold of detection of 7.8 pg/ml (•, solid line). Jurkat cells stably expressed ZSG1 (Δ, dotted line, right *y* axis) or NB-ZSG1 (•, dotted line, right *y* axis). Representative data from six independent experiments with similar results are shown. The symbol † indicates experiment was ended. (B) Cell lysates made from Jurkat-NB-ZSG1 or Jurkat-ZSG1 cells on the days shown were assayed by Western blotting with an anti-Tat (top panel), anti-tubulin (bottom panel), or anti-HIV-1 Gag antibody. (C) Total cellular RNA was isolated from Jurkat-ZSG1, Jurkat-NB-ZSG1, and Jurkat cells. HIV-1 mRNA was measured by qRT-PCR, and the values were normalized to the levels of endogenous GAPDH mRNA in the sample. A plus sign indicates that HIV-1 mRNA was not detected in the sample. Representative data from six independent experiments with similar results are shown. The RT-PCR assays were performed three times in triplicate. The data presented are mean values ± the standard deviations.

We tested the antiviral activity of stably expressed NB-ZSG1 by using 4 × 10^5^ Jurkat-NB-ZSG1, Jurkat-ZSG1, or nontransduced Jurkat cells that were infected with HIV-1_NL43_ with viral supernatant containing 20 ng of capsid (CA). Virus replication in Jurkat and Jurkat-ZSG1 cells was evident by detecting HIV-1 CA in culture supernatant, but no measurable HIV-1 CA was detected in supernatant from Jurkat-NB-ZSG1 cells over the 64-day experiment (Fig. 1A). The same result was obtained in six independent experiments. The level of NB-ZSG1- or ZSG1-positive cells remained >95% in both Jurkat cell lines (Fig. 1A), and NB-ZG1 expression was confirmed by Western blotting with an anti-Tat antibody (Fig. 1B). HIV-1 pr55^Gag^ was detected by Western blotting only in HIV-1-infected Jurkat and Jurkat-ZSG1 cells and not in HIV-1-infected Jurkat-NB-ZSG1 cells (Fig. 1B). Finally, we isolated total RNA from uninfected Jurkat cells or HIV-1-infected Jurkat, Jurkat-NB-ZSG1, and Jurkat-ZSG1 cells and performed an RT-PCR assay to measure viral RNA in the 5′-R-U5 region that is present on all spliced and unspliced viral RNA (Fig. 1C). We detected viral RNA in Jurkat and Jurkat-ZSG1 cells collected on day 3, and a >2-log higher viral RNA level was measured on day 28. No viral RNA was detected in Jurkat-NB-ZSG1 cells collected on the same days or in uninfected Jurkat cells. We conclude that stable expression of NB-ZSG1 can prevent HIV-1 replication in Jurkat cells.

### HIV-1 can infect Jurkat-NB-ZSG1 cells.

**FIG 2:**
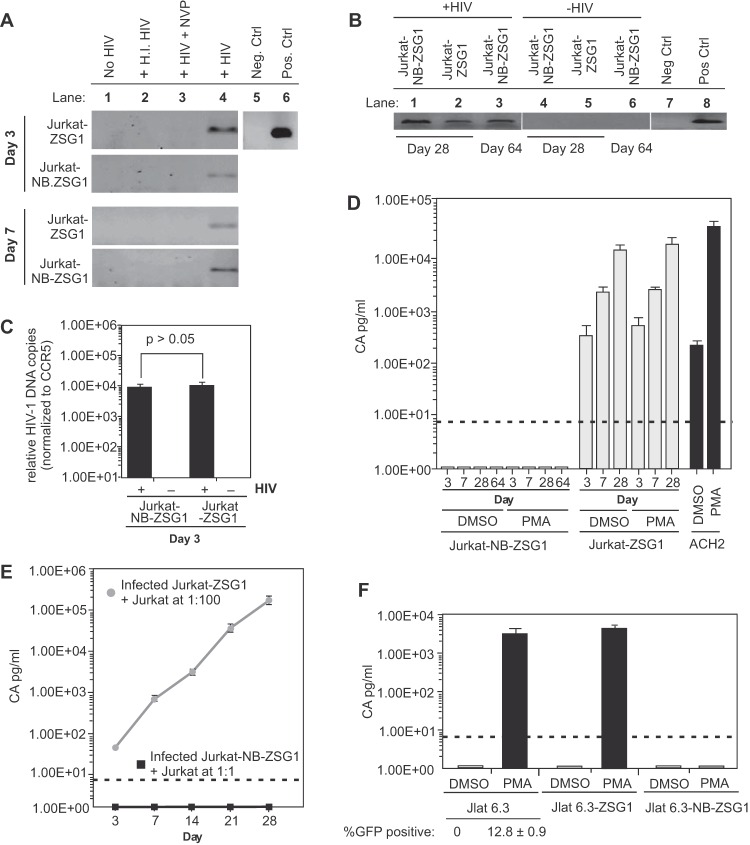
Jurkat-NB-ZSG1 cells harbor proviral HIV-1 DNA but no detectable viral mRNA. (A) PCR amplification of an HIV-1 *env* gene segment with total cellular DNA obtained from uninfected Jurkat, Jurkat-ZSG1, and Jurkat-NB-ZSG1 cells (lane 1) or from the same cell lines incubated with heat-inactivated (H.I.) virus (lane 2) or treated with or without nevirapine (NVP) for 2 h and infected with HIV-1_NL4.3_ (lanes 3 and 4). A PCR master mix alone was used as a negative (Neg.) control (lane 5) or with proviral DNA added as a positive (Pos.) control (Ctrl.) (lane 6). (B) Total cellular DNA obtained on days 28 and 64 from HIV-1-infected cell lines and assayed by endpoint PCR for *env* DNA. No viral DNA was detected in uninfected cells processed simultaneously (lanes 4 to 6). Negative and positive controls as in panel A are shown (lanes 7 and 8). (C) Total cellular DNA from day 3 HIV-1-infected or uninfected Jurkat-NB-ZSG1 and Jurkat-ZSG1 cells was assayed by *Alu*-PCR for integrated proviral DNA. The relative copy number was normalized to the CCR5 gene level in each sample. (D) On the days indicated, 1 nM PMA or DMSO (carrier) was added to Jurkat-NB-ZSG1 and Jurkat-ZSG1 cell cultures and incubated for 24 h. The soluble CA in the culture supernatant was then quantified by ELISA (the dotted line shows the limit of detection). ACH2 cell were used as a control for activation of HIV-1 gene expression by PMA. (E) Coculture of HIV-1-infected Jurkat-NB-ZSG1 or Jurkat-ZSG1 cells with uninfected Jurkat cells at a ratio of 1:1 or 1:100, respectively, for 28 days. The CA level in the supernatant was assayed by ELISA. (F) JLat 6.3, JLat 6.3-ZSG1, and JLat 6.3-NB-ZSG1 cells were incubated with 10 nM PMA for 24 h, and the CA levels in the supernatants were assayed by ELISA (the dotted line shows the limit of detection). The GFP-positive cell population was measured by flow cytometry. The assays in panels E and F were performed three times in triplicate, and the mean values and standard deviations are shown. Experiments representative of at least three independent experiments with similar results are shown. The *P* value in panel C was calculated with a Student *t* test.

One possibility for replication inhibition by NB-ZSG1 is that HIV-1 failed to enter, reverse transcribe, or integrate proviral DNA in Jurkat-NB-ZSG1 cells. To test this possibility, total cellular DNA was isolated from uninfected or HIV-1-infected Jurkat, Jurkat-ZSG1, and Jurkat-NB-ZSG1 cells sampled periodically and assayed for HIV-1 DNA by PCR. The results clearly show that viral DNA was present in all of the cell lines on days 3 and 7 postinfection but not in uninfected cells, cells incubated with heat-inactivated HIV-1, or cells treated with the reverse transcriptase inhibitor nevirapine (Fig. 2A), and viral DNA in Jurkat-NB-ZSG1 cells was detected up to 64 days postinfection when the experiment was ended (Fig. 2B). *Alu*-PCR analysis of chromosomal DNA obtained from infected Jurkat-NB-ZSG1 and Jurkat-ZSG1 cells 3 days postinfection indicated no significant difference between the relative levels of integrated proviral DNA in the two infected cell lines (Fig. 2C). We conclude that Jurkat-NB-ZSG1 cells are infected by HIV-1 but do not support viral replication.

### Latency-reversing agents (LRAs) do not rescue HIV-1 proviral DNA expression in Jurkat-NB-ZSG1 cells.

We performed experiments to determine if viral gene expression or replication could be rescued by exposing cells to phorbol ester 12-myristate 13-acetate (PMA), which stimulates NF-κB activity and can stimulate HIV-1 gene expression in productively and latently infected cells (26). HIV-1-infected Jurkat-ZSG1 and Jurkat-NB-ZSG1 cells were stimulated with 1 nM PMA. No measurable HIV-1 CA was detected (threshold of detection, 7.8 pg/ml) following the addition of PMA to all Jurkat-NB-ZSG1 samples (Fig. 2D). However, PMA efficiently induced a >2-log increase in HIV-1 CA production in the latently HIV-1-infected cell line ACH2 (Fig. 2D). We also confirmed that PMA upregulated T cell activation markers CD25 and CD69 on Jurkat-NB-ZSG1 and Jurkat-ZSG1 cells similarly (see Fig. S2A in the supplemental material), indicating that downregulation of HIV-1 gene expression was specific. Each cell line was also treated with JQ1 (a thienotriazolodiazepine and a potent inhibitor of the bromodomain and extraterminal motif family of bromodomain proteins) or suberanilohydroxamic acid (SAHA [also called vorinostat]). JQ1 allows Tat recruitment of SEC to stimulate HIV-1 elongation (27), while SAHA is a pan-histone deacetylase (HDAC) inhibitor (see Fig. S2B). Only SAHA induced CA production by Jurkat-NB-ZSG1 cells to detectable levels, which were still >300-fold lower than the levels of CA produced by drug-treated Jurkat-ZSG1 cells. We conclude that the HIV-1 provirus in Jurkat-NB-ZSG1 cells cannot be reactivated to a detectable level by PMA or JQ1, but SAHA can modestly stimulate HIV-1 gene expression, suggesting that NB-ZSG1 expression in Jurkat cells leads to histone modifications that are opposed by SAHA.

### Cell-to-cell transmission of HIV-1 by Jurkat-NB-ZSG1 cells to uninfected Jurkat cells is not detected.

Coculture experiments were performed to determine if HIV-1 produced below detection limits by Jurkat-NB-ZSG1 cells could infect Jurkat cells. HIV-1-infected Jurkat-NB-ZSG1 cells were mixed with uninfected Jurkat cells at a 1:1 ratio, while HIV-1-infected Jurkat-ZSG1 cells were cocultured with Jurkat cells at a 1:100 ratio as a control. HIV-1 replication was not detectable in the Jurkat-NB-ZSG1 cell coculture, whereas robust HIV-1 replication was detected in Jurkat-ZSG1 cell cocultures (Fig. 2E). We conclude that although Jurkat-NB-ZSG1 cells harbor HIV-1 provirus, they are unable to produce transmissible HIV-1 capable of infecting Jurkat cells.

### NB-ZSG1 inhibits activation of HIV in JLat 6.3 cells stimulated with LRAs.

The Jurkat latent 6.3 (JLat 6.3) cell line is a cloned Jurkat cell line that contains a latently integrated HIV-1 green fluorescent protein (GFP) reporter provirus, where epigenetic factors and DNA CpG methylation are implicated in silencing (28). JLat 6.3 cells do not produce detectable GFP or exhibit viral CA expression, but both proteins can be induced by compounds such as PMA (29). Therefore, JLat 6.3 cells were transduced with VLPs conveying NB-ZSG1 or ZSG so that >95% of the cell population expressed the introduced protein. Subsequently, parental JLat 6.3 cells, as well as JLat 6.3-NB-ZSG1 and JLat 6.3-ZSG1 cells, were incubated for 24 h in a medium containing 10 nM PMA and the amount of CA present in the culture supernatant was measured by enzyme-linked immunosorbent assay (ELISA). We confirmed that exposure of JLat 6.3 cells to PMA increased GFP expression from undetectable levels to ~12.8% of the cells in the JLat 6.3 cell population (Fig. 2F; see Fig. S3A in the supplemental material). In repeated experiments, PMA-stimulated JLat 6.3 and JLat 6.3-ZSG1 cells produced 2 to 4 ng/ml of CA, whereas CA was not detected in supernatant produced by JLat 6.3-NB-ZSG1 cells (Fig. 2F). All JLat 6.3 cell lines were incubated with the LRA JQ1 or SAHA for 24 h (see Fig. S3B in the supplemental material). Only SAHA increased HIV-1 CA expression in JLat 6.3-NB-ZSG1 cells to a detectable but low level, while JQ1 and SAHA strongly increased CA levels in Jurkat-ZSG1 and parental Jurkat cells (see Fig. S3B). We also incubated ACH2 cells that stably expressed NB-ZSG1 or ZSG1 with the same compounds. ACH2 cells harbor an HIV-1 provirus with a point mutation (C37→T) in TAR that impairs the activation of HIV-1 transcription by Tat. Like JLat 6.3 cells, ACH2 cells expressing NB-ZSG1 were strongly resistant to HIV-1 activation by PMA, JQ1, and SAHA, unlike parental ACH2 cells or ACH2 cells expressing ZSG1 (see Fig. S4 in the supplemental material). The data suggest that Nullbasic can strongly downregulate HIV-1 gene expression by mechanisms not involving transactivation by Tat via the TAR RNA–P-TEFb axis. The combined results show that NB-ZSG1 can inhibit activation of HIV transcription in JLat 6.3 and ACH2 cells in the presence of PMA, JQ1, and SAHA.

### NB-ZSG1 can attenuate HIV-1 production in productively infected Jurkat cells.

**FIG 3:**
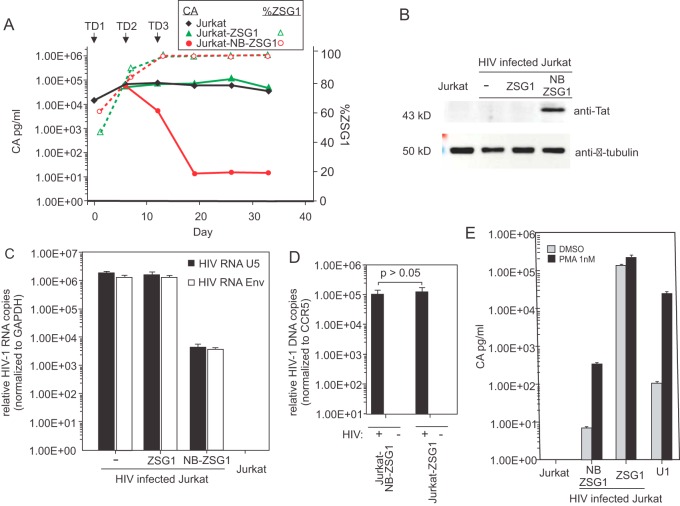
Postinfection treatment of HIV-1-infected Jurkat cells with NB-ZSG1 VLPs inhibits HIV-1 gene expression. (A) Jurkat cells were infected with HIV-1 and cultured for 30 days. The infected cells were transduced with VLPs conveying NB-ZSG1 or ZSG1 so that >95% of the cells were positive for ZSG1 or NB-SZG1 (three successive transductions [TD1, TD2, and TD3], as shown). HIV-1 CA in supernatant from HIV-1-infected Jurkat cells or NB-ZSG1-treated or ZSG1-treated, HIV-1-infected Jurkat cells was quantified by ELISA (left *y* axis, solid lines) or NB-ZSG1 and ZSG1 expression level in cells by flow cytometry (right *y* axis, dotted lines). An experiment representative of at least three independent experiments with similar results is shown. (B) Western blot analysis of the cell lysates from panel A with anti-Tat and anti-β-tubulin antibodies. The molecular masses of the NB-ZSG1 fusion protein and β-tubulin are indicated. (C) Total cellular RNA was isolated from HIV-1-infected Jurkat; NB-ZSG1-treated or ZSG1-treated, HIV-1-infected Jurkat; or uninfected Jurkat cells. qRT-PCR assays were performed with primers specific for the 5′ UTR or for sequences in *env*. The relative level of viral 5′ UTR RNA in each sample was normalized to the amount of GAPDH mRNA in the sample. (D) Total cellular DNA was isolated and subjected to *Alu*-PCR analysis for the cell lines. In panels C and D, mean values and standard deviations from experiments representative of three independent experiments performed in triplicate are shown. A two-tailed Student *t* test with equal variance was used to calculate the *P* value. (E) PMA stimulation of HIV-1 gene expression in an NB-ZSG1-treated, HIV-1-infected Jurkat cell population. Uninfected Jurkat cells, HIV-1-infected Jurkat cells treated with ZSG1 or NB-ZSG1, and U1 cells were incubated with 1 nM PMA for 24 h, and the concentration of CA in the supernatant was measured by ELISA. The experiment was performed in triplicate and repeated three times with similar results. The mean values and standard deviations of a representative experiment are shown.

We next examined if constitutively expressed NB-ZSG1 could inhibit HIV-1 in chronically and productively infected Jurkat cells. To test this, we infected Jurkat cells with HIV-1 and cultured them for 30 days; these cells stably produced HIV-1. The HIV-1-infected Jurkat cells were transduced with VLPs conveying genes encoding NB-ZSG1 or ZSG1. Because of a facility restriction, we were unable to select HIV-1-infected transduced cells by FACS. Instead, serial transductions were performed to obtain cell populations in which >95% of the cells expressed NB-ZSG1 or ZSG1. As shown in Fig. 3A, the first transduction (TD1) with NB-ZSG1 VLPs (day 0) did not affect CA production compared to that in infected control cells (day 6). A second transduction (TD2) on day 6 with NB-ZSG1 VLPs resulted in a 30-fold decrease in HIV-1 CA production (day 12), and a third transduction (TD3) on day 12, which resulted in >95% of the cells expressing NB-ZSG1, resulted in a >3-log reduction in HIV-1 CA production (day 20). Transduction with ZSG1 VLP had no effect on CA production. NB-ZSG1 expression was confirmed by Western blotting with an anti-Tat antibody (Fig. 3B). The results show that NB-ZSG1 can strongly suppress HIV-1 production in chronically infected Jurkat cells.

### NB-ZSG1 reduces HIV-1 mRNA levels in chronically infected Jurkat cells.

The relative numbers of HIV-1 RNA copies (normalized to glyceraldehyde 3-phosphate dehydrogenase [GAPDH] mRNA) were measured in the transduced cell lines, the nontransduced infected Jurkat cells, and uninfected Jurkat cells by quantitative RT-PCR (qRT-PCR) with primers targeting the U5 region (upstream of the 5′ major splice donor site) of the LTR shared by all HIV-1 mRNAs and a region in the HIV-1 *env* gene. The results obtained with both PCR primer pairs indicated a >2.5-log reduction in HIV-1 RNA in NB-ZSG1-treated Jurkat cells compared to the levels in untreated infected Jurkat and ZSG1-treated, HIV-1-infected Jurkat cells (Fig. 3C), which correlated with reduced HIV-1 production measured by CA ELISA of cell culture supernatant (Fig. 3A). Transcriptome sequencing (RNA-seq) analysis of the same RNA samples confirmed a >2-log decrease in the full-length HIV-1 RNA made by NB-ZSG1-treated, HIV-1-infected Jurkat cells (see Fig. S5 in the supplemental material) compared to that made by ZSG1-treated and untreated Jurkat cells. We measured the level of proviral DNA in the NB-ZSG1-treated and ZSG1-treated Jurkat cells by *Alu*-PCR. As shown in Fig. 3D, there was no significant change in the level of proviral DNA present in the cell lines. We next tested if PMA could stimulate HIV-1 transcription and gene expression in NB-ZSG1-treated, HIV-1-infected Jurkat cells. We incubated the same three cell lines in 1 nM PMA and measured the CA levels in the supernatant after 24 h. PMA increased CA production in NB-ZSG1-treated, HIV-1-infected Jurkat cells from 7 to >300 pg/ml, a 44-fold increase (Fig. 3E). A 2.25-fold increase in CA production (>200 ng/ml) was observed in ZSG1-treated, HIV-1-infected Jurkat cells, whereas CA production by latently infected U1.1 cells was induced by >200-fold. The outcome indicates that Nullbasic arrests HIV-1 transcription but it can be partially restored by PMA treatment of the NB-ZSG1-treated, HIV-1-infected Jurkat cell population.

### Knockdown of NB-ZSG1 expression rescues HIV-1 production in treated cells.

**FIG 4:**
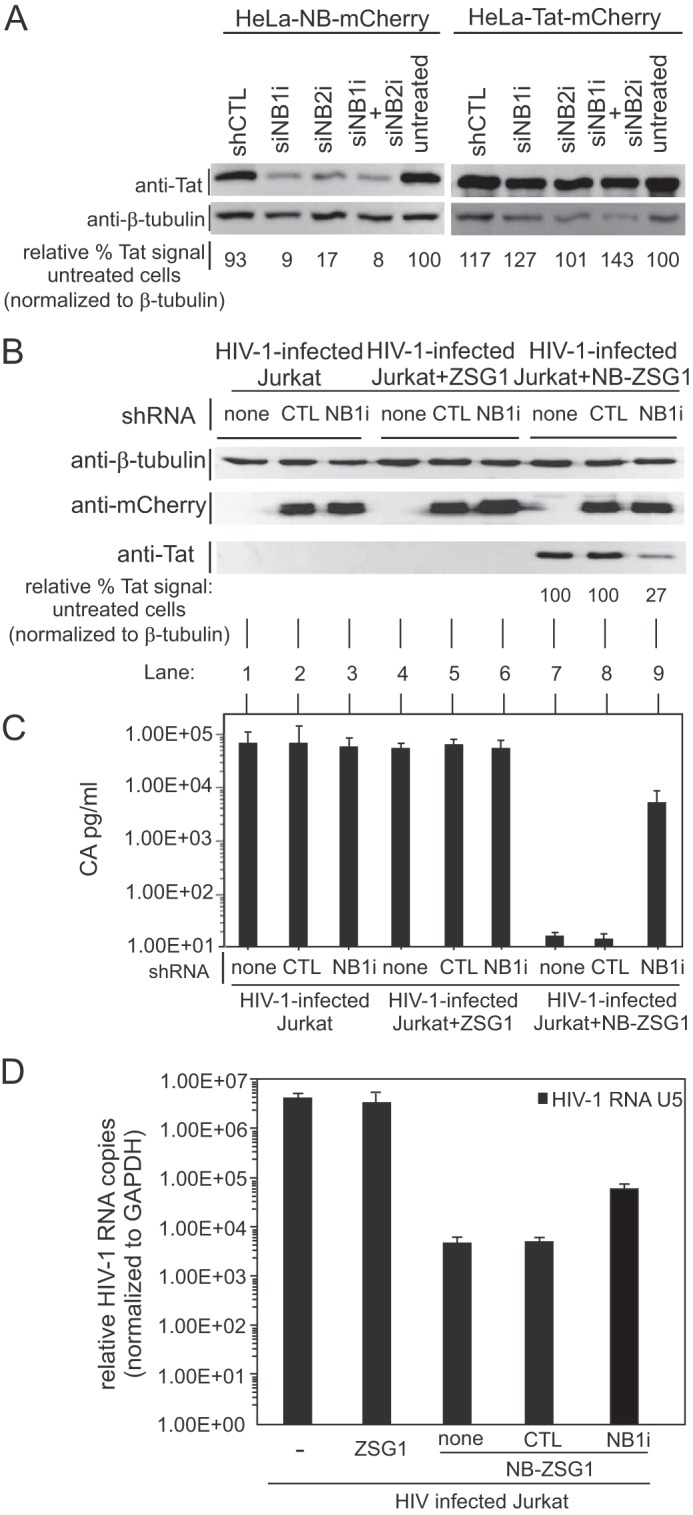
Virus production is restored in NB-ZSG1-treated, HIV-1-infected Jurkat cells by knockdown of NB-ZSG1. (A) Stable HeLa cell lines expressing NB-mCherry or Tat-mCherry were transfected alone or together with two siRNAs (siNB1i and siNB2i) or with a scrambled negative-control (shCTL) siRNA sequence. Cell lysates were analyzed by Western blotting with anti-Tat and anti-β-tubulin antibodies as indicated. A digital image of each Western blot assay was processed with ImageJ software, and the relative Tat signal level was normalized to the β-tubulin signal level in the same sample. (B) Lentiviral vectors that expressed shRNA based on siNB1i or siCTL sequences or the siCTL sequences were used to transduce Jurkat-NB-ZSG1 cells as indicated. Cell lysates were prepared from transduced cells and probed by Western blotting with anti-Tat, anti-mCherry, and anti-β-tubulin antibodies. The relative Tat signal in each sample was measured as described for panel A. (C) The HIV-1 CA present in culture supernatant was quantified by ELISA. Mean values and standard deviations from three independent experiments are shown. (D) Total cellular RNA was isolated from HIV-1-infected Jurkat; NB-ZSG1- or ZSG1-treated, HIV-1-infected Jurkat; or uninfected Jurkat cells. qRT-PCR assays were performed with primers specific for the 5′ UTR. The relative level of viral 5′ UTR RNA in each sample was normalized to the amount of GAPDH mRNA in the sample.

To further confirm that expression of Nullbasic is responsible for decreased CA production in infected Jurkat cells, we designed two small interfering RNA (siRNAs), siNB1i and siNB2i, that specifically target NB-ZSG1 mRNA for degradation but do not affect wild-type Tat. To validate the effectiveness and specificity of the siRNAs, stable HeLa cell lines expressing Tat-mCherry or NB-mCherry were transfected with the siRNAs individually or in combination. A scrambled siRNA (siCRTL) was used as a control. As shown in Fig. 4A, Tat-mCherry levels in HeLa cells were unaffected by either siRNA, whereas the amount of NB-mCherry in cells was reduced by >80% when it was used individually or in combination.

Because Jurkat cells are refractory to common transfection methods, a lentiviral vector expressing a short hairpin RNA (shRNA) based on NB1i was made (shNB1i) and delivered to the NB-ZSG1-treated, ZSG1-treated, and untreated, HIV-1-infected Jurkat cell lines (Fig. 4B). We also used a lentiviral vector to deliver shCTRL, a scrambled-sequence negative-control shRNA, to the same Jurkat cell lines. The lentiviral vector used coexpressed mCherry to enable monitoring of shRNA expression. As shown in Fig. 4B, shNB1i reduced NB-ZSG1 levels by >70% compared to those in nontransduced Jurkat-NB-ZSG1 cells (compare lane 9 to lane 7), whereas shCTRL had no effect on NB-ZSG1 expression in cells (compare lane 9 to lane 8). The reduced level of NB-ZSG1 in cells resulted in a >300-fold CA production increase, compared to that in cells expressing shCTRL (Fig. 4C, compare lane 9 to lane 8) or the original Jurkat-NB-ZSG1 cells (Fig. 4C, compare lane 9 to lane 7), and increased viral mRNA (Fig. 4D), consistent with increased transcription from the HIV-1 LTR promoter. The shNB1i and shCTRL did not affect virus production in HIV-infected or ZSG1-treated Jurkat cells (Fig. 4C, lanes 1 to 6). These outcomes indicate that inhibition of HIV-1 production is due to the expression of NB-ZSG1. This result is also in line with previous reports that Nullbasic inhibits HIV-1 gene expression in a dose-dependent manner (20).

### NB-ZSG1 inhibits RNAPII association with the LTR promoter and suppresses acetylation of histone 3 lysine 9.

**FIG 5:**
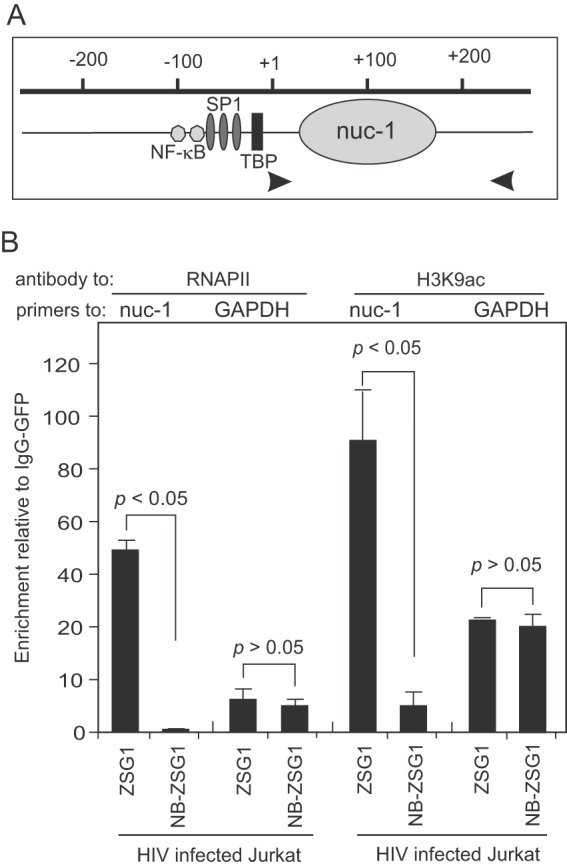
ChIP assays for a DNA-protein complex in HIV-1-infected Jurkat cells treated with ZSG1 or NB-ZSG1. (A) Schematic of the HIV-1 LTR promoter from −200 to +200. The binding sites for NF-κB, SP1, and the TATA binding protein (TBP) and the location of *nuc-1* are indicated. Viral DNA in the U5 region was detected with primers specific for HIV_NL43_ (indicated by arrowheads). (B) ChIP assays were performed with an anti-RNAPII or anti-H3K9ac antibody and primers for *nuc-1*. The gene for GAPDH was used as a reference. The relative enrichment of viral DNA in the immunoprecipitation reaction was calculated as a signal over the background with the same DNA-protein complex and an isotype-matched anti-GFP antibody. The experiments were performed three times, and the mean values and stand deviations are shown. The *P* values were calculated with a Student *t* test.

The sharp decrease in viral RNA and protein levels suggested that Nullbasic may affect the association of RNAPII with the LTR, phosphorylation of RNAPII, or the acetylation state of HIV-1 Nuc1. First, we performed chromatin immunoprecipitation (ChIP) assays of protein-DNA complexes from cell lysates made from NB-ZSG1-treated, HIV-1-infected Jurkat cells or ZSG1-treated, HIV-1-infected Jurkat cells with an anti-RNAPII antibody or an isotype-matched anti-GFP negative-control antibody. The captured protein and DNA complexes were probed with primers that targeted the HIV-1 U5 region encompassing *nuc-1* (Fig. 5A). The results showed the occupancy of RNAPII on *nuc-1* in NB-ZSG1-treated, HIV-1-infected Jurkat cells to be very low and near the level of sensitivity of the ChIP assay, whereas a 48-fold enrichment of RNAPII occupancy on *nuc-1* was measured in samples prepared from ZSG1-treated, HIV-1-infected Jurkat cells (Fig. 5B). We also measured the level of acetylated histone 3 lysine 9 (H3K9ac), which is a marker of HIV-1 LTR promoter activation, in the same cell lysate samples (Fig. 5B). The results showed a large reduction in the levels of H3K9ac in NB-ZSG1-treated, HIV-1-infected Jurkat cells compared to those in ZSG1-treated, HIV-1-infected Jurkat cells, suggesting that NB-ZSG1 inhibits HIV-1 transcription by mechanisms that include epigenetic changes to chromatin. The occupancy of RNAPII or the level of H3K9ac on the GAPDH open reading frame was unchanged by NB-ZSG1, demonstrating that the changes are specific to the HIV-1 LTR promoter (Fig. 5B). These outcomes correlate to the sharply lower levels of viral RNA in NB-ZSG1-treated cells (Fig. 5A and B). The data indicate that NB-ZSG1 expression in Jurkat cells reduced the association of RNAPII with the HIV-1 LTR promoter because of a mechanism(s) that includes epigenetic changes to HIV-1 chromatin.

## DISCUSSION

HIV-1 Tat is a master regulator of HIV-1 gene expression and latency (18). Tat plays a direct role in the activation of HIV-1 transcription by RNAPII by recruitment of P-TEFb to the HIV-1 promoter via *tar* RNA. In addition, Tat regulates viral replication by alternative mechanisms, including chromatin remodeling (30–32), mRNA splicing (33), Rev function (23), and microRNA biogenesis (34, 35). In this paper, we show that a mutant Tat protein, Nullbasic, can strongly antagonize wild-type Tat activity and suppress transcription by up to hundreds-fold in Jurkat and ACH2 cells. Our data show that Nullbasic, in addition to directly targeting P-TEFb (20, 24), is able to affect epigenetic modifications of histone proteins on the HIV-1 LTR promoter and inhibit the association of RNAPII with the HIV-1 LTR. We conclude that expression of Nullbasic in HIV-1-infected Jurkat cells leads to transcriptionally silent proviral DNA. Downregulation of Nullbasic levels in cells can restore virus production, indicating that Nullbasic is responsible for the induction of an HIV-1 “silent” state in Jurkat cells.

We tested the effects of Nullbasic on HIV-1 replication in three scenarios, in which Nullbasic was introduced into uninfected Jurkat cells and then they were infected with HIV-1, Nullbasic was introduced into latently infected JLat 6.3 or ACH2 cells, or Nullbasic was introduced into Jurkat cells productively infected with HIV-1. In all cases, Nullbasic strongly inhibited HIV-1 replication and virus production. First, infection of Jurkat-NB-ZSG1 cells with HIV-1 resulted in measurable amounts of integrated proviral DNA but did not lead to productive infection in repeated experiments. No viral RNA was detected in these experiments, suggesting that HIV-1 transcription was inhibited in Jurkat-NB-ZSG1 cells. However, Nullbasic’s two additional antiviral activities, inhibition of Rev function and causing aberrant uncoating and defective RT (20, 22–24, 36), would also suppress viral spread in these experiments. Indeed, we could not recover infectious HIV-1 by stimulation with PMA or by coculture of uninfected Jurkat cells with HIV-1-infected Jurkat-NB-ZSG1 cells. Likewise JLat 6.3 cells expressing NB-ZSG1 could not produce measurable viral CA when stimulated with PMA, whereas parental JLat 6.3 and JLat 6.3 ZSG1 cells did. When HIV-1-infected Jurkat cells were transduced with an NB-ZSG1 VLP (Fig. 3A), a sharp >3-log decline in virion production was observed, which correlated with a 2.5-log decrease in mRNA present in the NB-ZSG1-treated, HIV-1infected Jurkat cells. The discrepancy between inhibition of viral protein and viral RNA levels could be caused by factors such as the anti-Rev activity of Nullbasic (20, 23, 24) and altered translation (37, 38). We favor the possibility that the CA detected in culture supernatant originated from HIV-1-infected Jurkat cells expressing no or low levels of NB-ZSG1. This possibility is supported by siRNA downregulation of Nullbasic that leads to increased CA production. Moreover, a large proportion of the Jurkat cells were productively infected with HIV-1, as indicated by high levels of CA present in the culture supernatant. Therefore, NB-ZSG1-negative and HIV-1-positive cells, which would include less than ~5% of the cells in the population, could account for the small amounts of CA detected in the culture supernatant. This possibility is supported by RT-PCR and RNA-seq analyses of viral mRNA obtained from chronically infected, NB-ZSG1-treated Jurkat cells that showed a sharply smaller amount of HIV-1 mRNA but underwent transcriptional elongation by RNAPII indicative of an active Tat–P-TEFb complex in the cells. We previously demonstrated that Nullbasic binds to P-TEFb (24) and can inhibit transaction of the HIV-1 LTR promoter by Tat as a dominant negative competitive inhibitor (20), as do other mutant Tat proteins (39–42). Therefore, we believe it is likely that the low level of viral transcripts is due to HIV-infected cells lacking sufficient levels of Nullbasic to completely inhibit transcription.

How Nullbasic is able to establish viral silencing remains to be determined. The mechanism could be related to mechanisms leading to HIV-1 latency in CD4^+^ T cells, which are only partly understood and regulated by multiple factors that include the genomic integration site of the HIV-1 provirus, the availability of transcription factors in the cell, regulation of chromatin structure by histone acetylation, and methylation and epigenetic changes in proviral DNA (16). As Nullbasic expression downregulated HIV-1 transcription in clonal (JLat 6.3 and ACH2) and nonclonal HIV-1-infected Jurkat cells, it is highly unlikely that proviral integration site selection accounts for decreased transcription. The large drop in RNAPII detected on the HIV-1 LTR is consistent with epigenetic alterations resulting in heterochromatin formation. Interestingly HIV-1 activation by LRAs in ACH2 cells, which have an HIV-1 provirus with a TAR mutation that is poorly responsive to transactivation by Tat, was strongly inhibited by NB-ZSG1. In HIV-1-infected Jurkat, JLat 6.3, and ACH2 cells expressing NB-ZSG1, the HDAC inhibitor SAHA could at least partially rescue HIV-1 transcription suggesting that NB-ZSG1 enhanced the deacetylation of *nuc-1* on the HIV-1 LTR. Learning how Nullbasic influences changes in *nuc-1* acetylation could provide clues to the silencing of HIV-1 therapeutically. Indeed, Nullbasic is a mutant Tat protein and the roles of wild-type Tat in chromatin remodeling and transactivation are diverse and complex. For example, HIV-1 Tat can recruit the epigenetic regulators SWI/SNF, p300, and C/EBP, which modulate the acetylation of *nuc-1* and HIV-1 transcription (1). Tat can be acetylated by p300/CBP and hGCN4, which regulates the Tat transactivation cycle (5). Tat can be methylated, which may affect interactions with cellular proteins (43–46). Tat also interacts with the transcription factor SP1, which is involved in chromatin remodeling and the transcriptional activity of the HIV-1 LTR promoter (47, 48). Our data suggest that Nullbasic disrupts important protein-protein interactions required for optimal chromatin remodeling and transcriptional activity, so that a determination of key interactions opposed by Nullbasic, which deserve further study, could inform strategies aimed at a functional cure for HIV infection.

A recently described Tat inhibitor, didehydro-cortistatin A (dCA), binds to Tat and strongly inhibits HIV-1 transcription in human cell line models of HIV-1 latency and in CD4 cells from virally suppressed subjects (49). The negative effects of dCA on HIV-1 transcription are similar to those of Nullbasic. Interestingly, dCA binds to the Tat ARM, while Nullbasic is a mutant Tat protein lacking an ARM, which could indicate that dCA and Nullbasic have similar mechanisms of action. Outcomes from our study highlight the importance of Tat in the regulation of HIV-1 activation and latency, a proposition also supported by a recent study implying a role for the Tat feedback loop in the regulation of the transition from latency independent of the cellular activation state (18, 50). In our study, Nullbasic could negate Tat feedback activation, resulting in a HIV-1 expression decrease to nondetectable or very low levels in infected Jurkat cells, as well as a decrease in viral gene expression in ACH2 cells less reliant on Tat transactivation. In conclusion, HIV-1 transcription in productively infected cells can be inhibited and possibly blocked by stable expression of Nullbasic. At the very least, Nullbasic can be used as a probe to interrogate protein-protein interactions, but its use may be amenable to a gene therapy approach able to provide durable protection of T cells from HIV-1 by inhibiting viral transcription and viral spread.

## MATERIALS AND METHODS

### Supplemental materials and methods.

For a detailed description of the supplemental materials and methods used in this study, see Text S1 in the supplemental material.

### HIV-1 stocks.

HIV-1_NL43_ stocks were made by transfection of HEK293T cells with a proviral DNA plasmid (51, 52). The medium was replaced 24 h posttransfection, and HIV-1 was harvested at 48, 72, and 96 h posttransfection. All virus stocks were quantified by CAp24 ELISA and stored in small aliquots at −80°C.

### Construction of a Nullbasic expression vector.

NB-ZSG1 and ZSG1 gene fragments were excised from pGCsamEN-NB-ZSG1 and pGCsamEN-ZSG1 (21) with NheI and EcoRI and inserted into a pSicoR-EF1a vector (Addgene, Cambridge, MA) at these restriction enzyme sites. The vector constructions were confirmed by DNA sequencing.

### VLP production.

VLPs that convey NB-ZSG1 or ZSG1 were produced by cotransfecting 3 µg of pSicoR-NB-ZSG1 or pSicoR-ZSG1 with 10 µg of plasmid pCMVΔR8.91 and 3 µg of pCMV-VSV-G into HEK293T cells with X-tremeGENE HP DNA transfection reagent (Roche Diagnostics GmbH, Mannheim, Germany) in accordance with the manufacturer’s protocol. The cell medium was replaced at 24 h posttransfection. VLPs were harvested by filtration with a sterile 0.2-µm syringe filter (Sartorius Stedim Biotech GmbH, Göttingen, Germany) at 48 h posttransfection. The concentration of VLPs was quantified with an HIV-1 CAp24 ELISA.

### CAp24 ELISA.

An HIV-1 CAp24 ELISA (Zeptometrix Corp., Buffalo, NY) was used to measure CA levels in culture supernatants in accordance with the manufacturer’s instructions.

### VLP titers.

NB-ZSG1 is packaged in VLPs, where it inhibits RT in the target cell (22). Therefore, the titers of all VLP stocks were determined by transduction of HEK293T cells with serial dilutions of each VLP stock. One transduction unit (TDU) is defined as the volume of a VLP supernatant required to transduce 10% of HEK293T cells. The percentage of ZSG1- or NB-ZSG1-positive cells at 48 h posttransduction was monitored by flow cytometry.

### Cell transduction.

A total of 2 × 10^5^ cells were transduced with 20 TDUs of NB-ZSG1 VLPs or ZSG1 VLPs in the presence of 8 µg/ml of hexadimethrine bromide (Sigma-Aldrich). For Jurkat cells, at 4 days posttransduction, ZSG1-positive cells were sorted with a MoFlo High Speed Cell Sorter (Beckman Coulter, Pasadena, CA). For HIV-1-infected Jurkat, JLat 6.3, and ACH2 cells, multiple transductions were performed to obtain >95% ZSG1-positive cells, except for ACH2 cells expressing NB-ZSG1, which were >81% positive. The purity of the sorted HIV-1-infected Jurkat, JLat 6.3, and ACH2 cells was measured by flow cytometry.

### Western blot analysis.

Cells were lysed in cell lysis buffer (20 mM Tris-HCl [pH 7.5], 150 mM NaCl, 1 mM Na_2_EDTA, 1% Triton, 2.5 mM sodium pyrophosphate, 1 mM β-glycerophosphate, 1 mM Na_3_PO_4_, 1 µg/ml leupeptin, 1 mM phenylmethylsulfonyl fluoride), and the supernatant produced by centrifugation at 16,000 × *g* for 15 min at 4°C was collected. Protein concentrations were measured with the Bio-Rad protein assay (Bio-Rad, Inc.). Thirty micrograms of total protein was used for Western blot assays. Different viral and cellular proteins were detected with rabbit anti-Tat (Diatheva, Fano, Italy), goat anti-HIV-1 Gag (Santa Cruz Biotechnology, Inc., Dallas, TX), mouse anti-β-tubulin (Sigma-Aldrich, St. Louis, MO), and rabbit anti-mCherry (Abcam, Cambridge, United Kingdom) antibodies. The primary antibodies were detected with horseradish peroxidase-conjugated goat anti-mouse or anti-rabbit antibodies (Thermo Fisher) as required.

### HIV-1 infection of Jurkat-NB-ZSG1 cells.

Jurkat-NB-ZSG1, Jurkat-ZSG1, and Jurkat cells (4 × 10^5^) were infected with HIV-1_NL4.3_ supernatant containing 20 ng of CA at 37°C for 2 h. The cells were washed three times with 1× phosphate-buffered saline (PBS) buffer and then cultured in 5 ml of RF10 in a 25-cm^2^ flask. Culture medium and cell samples were collected on days 3, 7, 10, 14, 17, 21, 24, and 28 for all three cell lines (Jurkat, Jurkat-ZSG1, and Jurkat-NB-ZSG1). The sampling period was 64 days for Jurkat-NB-ZSG1 cells. The samples were collected weekly after day 28. Cells were collected by centrifugation at 300 × *g* for 10 min. The cell pellets were washed three times with 1× PBS buffer and then used for cell flow and DNA, RNA, and protein extraction. The culture medium was collected, and CA levels were measured with an HIV-1 CAp24 ELISA.

### HIV-1 PCR and *Alu* assay.

HIV-1_NL4.3_ stock was treated with DNase I to eliminate the proviral DNA. As negative controls for the experiment, HIV-1_NL4.3_ was heat inactivated at 90°C for 30 min or cells were treated with 500 µM nevirapine for 2 h prior to infection. Jurkat, Jurkat-ZSG1, and Jurkat-NB-ZSG1 cells (4 × 10^5^) were infected with HIV-1_NL4.3_ with or without 500 µM nevirapine or with heat-inactivated HIV-1_NL4.3_. Cell samples were collected on days 3, 7, 28, and 64 postinfection. Genomic DNA was extracted with the QIAamp genomic DNA kit (Qiagen, Hilden, Germany). Viral DNA was detected by PCR with forward primer 5′-AGCTCATCAGAACAGTCAG and reverse primer 5′-CAGAGTGGGGTTAATTTTAC, which are specific for HIV-1_NL4.3_. Amplification was carried out for 1 cycle of 95°C for 2 min; 35 cycles of 95°C for 20 s, 50°C for 20 s, and 72°C for 20 s; and 1 cycle of 72°C for 7 min. *Alu-*PCR was performed as described previously (53), with slight modifications of the primers used because of DNA sequence similarities between the pSicoR vector and HIV-1_NL4.3_. Briefly, *Alu-*PCR was performed with Fast Start TaqMan master mix (2×; Roche, Basel, Switzerland). Five hundred nanograms of genomic DNA of each sample was used for a first-round PCR in a 50-µl reaction volume with HIV-1 first-strand forward primer 5′-GGTCTCTCTGGTTAGACCAA and *Alu* reverse primer 5′-TGCTGGGATTACAGGCGTGAG. The PCRs underwent 1 cycle of 95°C for 10 min; 25 cycles of 95°C for 30 s; 52°C for 20 s, and 72°C for 2 min; and 1 cycle of 72°C for 7 min. A 5-µl volume of the first PCR product was then used in a nested-PCR protocol. A nested PCR was performed with HIV-1 forward primer 5′-AACTAGGGAACCCACTGCTTAAG, HIV-1 reverse primer 5′-TGGTTCTACTTTCGCTTTCGC, and TaqMan probe SL72 (5′-6-carboxyfluorescein–CGGTCGAGTGCTTCAAGTAGTGTGTGCCCGTCCGACCG–6-carboxytetramethylrhodamine). The reaction conditions were 1 cycle of 95°C for 2 min and 45 cycles of 95°C for 15 s and 60°C for 1 min. A TaqMan probe-based gene expression analysis assay for CCR5 was used to normalize each sample (Thermo Fisher).

### Coculture experiments.

HIV-1-infected Jurkat-NB-ZSG cells were cocultured with uninfected Jurkat cells at a ratio of 1:1. Infected Jurkat and Jurkat-ZSG1 cells were cocultured with uninfected Jurkat cells at a ratio of 1:100. Culture supernatant was collected on days 3, 7, 14, 21, and 28. The level of HIV-1 in each sample was determined by CAp24 ELISA.

### NB-ZSG1-transduced, HIV-1-infected Jurkat cells.

Transduced, HIV-1-infected Jurkat cells were grown for 30 days, and the supernatant was sampled periodically. RNA, DNA, and protein were isolated from the cells, and CA levels in the culture supernatant were measured by ELISA. The RNA samples were used for RNA-seq and real-time PCR analysis. The DNA samples were used for *Alu-*PCR analysis, and the protein samples were used for Western blotting.

### PMA stimulation.

HIV-1-infected Jurkat, Jurkat-ZSG1, Jurkat-NB-ZSG1, ACH2, and U1 cells (1 × 10^6^) were stimulated with 1 nM PMA or the 0.1% dimethyl sulfoxide (DMSO) vehicle control for 24 h. Uninfected Jurkat, Jurkat-NB-ZSG1, and Jurkat-ZSG1 cells were used as negative controls for the assay. JLat 6.3, JLat 6.3-ZSG1, and JLat 6.3-NB-ZSG1 cells were simulated with 10 nM PMA or 0.1% DMSO for 24 h. Both supernatant and cell samples were collected from each treatment. The HIV-1 CA level in the culture supernatant was quantified by ELISA. GFP expression in parental JLat 6.3 cells was measured by flow cytometry.

### qRT-PCR assays.

RNA was isolated from cells with TRIzol reagent (Thermo Fisher, Waltham, MA) in accordance with the manufacturer’s protocol. All RNA samples were treated with Turbo DNase I and used in a PCR to confirm the removal of any contaminating HIV-1 DNA. cDNA was synthesized with 500 ng of total RNA, random hexamer primers, and Superscript III reverse transcriptase (Thermo Fisher, Waltham, MA) in accordance with the manufacturer’s instructions. HIV-1 RNA was quantified by real-time PCR with primers that targeted the HIV-1 5′-R-U5 region (forward, 5′-GGTCTCTCTGGTTAGACCA; reverse, 5′-TGGTTCTACTTTCGCTTTCGC) and envelope region (forward, 5′-GCATATGATACAGAGGTAC; reverse, 5′-CAGAGTGGGGTTAATTTTAC). SYBR green master mix (Scientifix, Cheltenham, Australia) was used for PCR. All HIV-1 copy numbers measured were normalized to the level of GAPDH mRNA in the same sample, which was measured by PCR with the following oligodeoxyribonucleotides: forward primer 5′-GCAAATTCCATGGCACCGTC and reverse primer 5′-TCGCCCCACTTGATTTTGG.

### ChIP.

Ten million NB-ZSG1- or ZSG1-treated, HIV-1-infected Jurkat cells were pelleted and washed once with 1× PBS buffer. The pellets were resuspended in 30 ml of 1× PBS buffer and cross-linked with 1% formaldehyde for 10 min at room temperature. The cross-link was stopped by adding glycine to a final concentration of 0.125 M. The cells were washed once with 1× PBS buffer and lysed in a buffer containing 1% SDS, 10 mM EDTA, 50 mM Tris (pH 8.0), and 1× protease inhibitor for 10 min on ice and then centrifuged at 350 × *g* for 5 min at 4°C. The supernatant was removed, and the cell pellet was resuspended in 0.8 ml of radioimmunoprecipitation assay buffer (1× PBS, 1% NP-40, 0.5% sodium deoxycholate, 0.1% SDS, 50 mM Tris [pH 8.0], 1 × proteinase inhibitor). Chromatin shearing was carried out in a 12- by 12-mm tube with a Covaris S220 sonicator (Covaris, Woburn, MA). The sonication conditions were 145 W and a 5% duty cycle for 8 min. Immunoprecipitation was performed with antibody-labeled Dynabeads (Thermo Fisher, Waltham, MA) at 4°C overnight; this was followed by four washes with wash buffer (100 mM Tris [pH 7.5], 500 mM LiCl, 1% NP-40, 1% Na-deoxycholate) prior to elution with a buffer containing 1% SDS and 0.1 M NaHCO_3_. The elute was reverse cross-linked overnight at 65°C, and the DNA was extracted with phenol-chloroform. The PCR targets the 5′ untranslated region (UTR) of the HIV-1 genome. The forward primer sequence is 5′-GGTCTCTGGTTAGACCA, and the reverse primer sequence is 5′-TGGTTCTACTTTCGCTTTCG. The results were analyzed by the fold enrichment method with the formula % enrichment = 2^−(*CT* IP − *CT* mock)^.

### Knockdown of NB-ZSG1 expression in Jurkat cells.

Two different siRNAs targeting Nullbasic were synthesized. The sequences are 5′-CAUCUCCUAUGGCGGUGGU (siNB1i) and 5′-UGGUCCUCCUCAAGGCAGU (siNB2i). To test the knockdown specificity and efficiency of the designed siRNAs, HeLa cells stably expressing either HIV-1 Tat or Nullbasic were transfected with siNB1i, siNB2i, and a universal negative-control siRNA (siCTRL) by using Lipofectamine RNAiMAX (Thermo Fisher, Waltham, MA) in accordance with the manufacturer’s instructions. Cells were incubated at 37°C for 72 h and then lysed in cell lysis buffer for Western blot analysis. A shRNA, shNB1i, that was based on the siNB1i sequence was constructed. An XbaI site was inserted into the sequence for screening, and the restriction sites HpaI and XhoI were added at the 5′ and 3′ ends, respectively. The shRNA fragments were ligated into pSicoR-Ef1a-mCh between the HpaI and XhoI sites downstream of a U6 promoter. The pSicoR.NBshRNA.mCh vector was confirmed by sequencing. VLPs containing shNB1i were made by cotransfecting HEK293T cells with 10 µg of plasmid pCMVΔR8.91, 3 µg of pCMV-VSV-G, and 3 µg of pSicoR.NBshRNA.mCh. Two rounds of transduction were performed, and flow cytometry showed that >90% of the cells were successfully transduced. The cells were grown for 72 h, after which cells were collected and a lysate was prepared as described previously. The culture supernatant was collected and analyzed for HIV-1 CA by ELISA.

### Statistical analysis.

Statistical analysis was performed with a two-tailed, paired Student *t* test from at least three independent experiments or measurements. Statistical significance was set at *P* < 0.05.

## SUPPLEMENTAL MATERIAL

Text S1  Supplemental materials and methods. Download Text S1, DOCX file, 0.01 MB

Figure S1  Transduction and stable expression of NB-ZSG1 or ZSG1 in Jurkat cells. (A) Representative flow cytometry analysis of nontransduced Jurkat cells (left) and FACS-isolated Jurkat-NB-ZSG1 (middle) and Jurkat-ZSG1 (right) cells. (B) Western blot analysis of NB-ZSG expression in Jurkat-NB-ZSG1, Jurkat-ZSG1, and nontransduced Jurkat cells. The cell lysates were probed by Western blotting with an anti-Tat (top) or anti-tubulin (bottom) antibody. The results shown are representative of experiments performed three times. (C) The proliferation and viability of all Jurkat cell lines were measured at 30 days posttransduction with the CellTiter 96 AQueous one solution cell proliferation (MTS) assay. The experiment was performed in triplicate, and mean values and standard deviations are shown. *P* values comparing outcomes with those obtained with Jurkat-ZSG1 and Jurkat-NB-ZSG1 cells were calculated with Student *t* tests. The experiment was repeated three times with similar results. Download Figure S1, EPS file, 0.5 MB

Figure S2  Activation of Jurkat cells infected with HIV-1. (A) Jurkat cells were incubated with PMA (1 nM), and after 24 h, they were stained with antibodies to T cell activation markers CD25 and CD69. The percentages of CD25- and CD69-positive cells are shown. All cell population were >95% positive for either NB-ZSG1 or ZSG1, as indicated. (B) Jurkat-NB-ZSG1, Jurkat-ZSG1, and ACH2 cells were incubated with JQ1, SAHA, or the DMSO vehicle control as indicated. After 24 h, the amount of CA in the culture supernatant was measured by ELISA. The dotted line indicates the detection limit of the ELISA. The experiment was performed in triplicate, and mean values and standard deviations are shown. An experiment representative of three experiments with similar results is shown. Download Figure S2, EPS file, 2.9 MB

Figure S3  Activation of JLat 6.3 cell lines. (A) JLat 6.3 cells stably expressing NB-ZSG1 or ZSG or cells of the parental line were incubated with JQ1, SAHA, or the DMSO vehicle control for 24 h, and the percentages of cells expressing ZSG1 or GFP (for parental JLat 6.3 cells only) were determined by flow cytometry. (B) The amount of CA present in the culture supernatant was measured by ELISA. The experiment was performed in triplicate, and mean values and standard deviations are shown. An experiment representative of three experiments with similar results is shown. Download Figure S3, EPS file, 2.9 MB

Figure S4  NB-ZSG1 inhibits activation of HIV-1 in ACH2 cells. ACH2 cells stably expressing NB-ZSG or ZSG1 or the cells of the parental line were incubated in JQ1, PMA, SAHA, or the DMSO vehicle control as indicated. After 24 h, the expression of NB-ZSG1 or ZSG1 was measured by flow cytometry. The amount of CA present in the culture supernatant was measured by ELISA. The experiment was performed in triplicate, and mean values and standard deviations are shown. An experiment representative of three experiments with similar results is shown. Download Figure S4, EPS file, 1.7 MB

Figure S5  An Integrative Genomics Viewer-generated coverage plot of the RNA-seq coverage of the HIV-1 isolate genome. Data were randomly resampled to the same library depth. The height of the plot directly indicates the number of times that nucleotide was observed in the data set. Colored bars indicate that the nucleotide observed was different from the nucleotide in the reference HIV-1 isolate genome. NB-ZSG1-treated, HIV-1-infected Jurkat cells (third panel) transcribed >2 log fewer virus molecules than ZSG1-treated, HIV-1-infected Jurkat cells (second panel) or untreated, HIV-1-infected Jurkat cells (first panel). However, the overall pattern of expression is similar (fourth panel), containing no obvious defects in splicing or transcription integrity. Download Figure S5, EPS file, 1.3 MB
