# Supplementary material for: Shutdown of HIV-1 Transcription in T Cells by Nullbasic, a Mutant Tat Protein
Source: mBio. 2016 Jul 5;7(4):e00518-16. doi: 10.1128/mBio.00518-16 (PMC4958243; doi:10.1128/mBio.00518-16)
Supplement: Text S1 — Supplemental materials and methods. Download [file mbo003162881s1.docx]

**Supplementary Methods**

**Chemical and reagents.** HEK293T and Jurkat cells were purchased from ATCC. Cell culture media, Dulbecco’s modiﬁed Eagle’s medium (DMEM), Roswell Park Memorial Institute 1640 medium (RPMI) penicillin/streptomycin, fetal bovine serum were purchased from Thermo Fisher (Waltham, MA). Chemicals are from Sigma-Aldrich (St. Louis, MI) unless mentioned.

**Cell culture maintenance.** HEK293T cells were grown in DMEM supplemented with penicillin (100U/ml), streptomycin (100 µg/ml), and 10% (v/v) FBS (referred as DF10). Jurkat cells were grown in RPMI 1640 medium with the same supplements as for DF10. All cells were grown at 37°C in a humidified 5% CO_2_ incubators.

**MTS assay.** The proliferation of Jurkat-NB-ZSG1 and Jurkat-ZSG1 cells were compared with Jurkat cells using CellTiter 96® AQueous One Solution Cell Proliferation assay (Promega, Madison, WI) according to the manufacturer’s instructions. Briefly, a 96-well plate was seeded with 5000 cells in 100 µl / well and incubated at 37°C for 72 h. Then 20 µl of Aqueous One Solution was added to each well and incubated at 37°C for 3 h and the colorimetric reaction was measured in plate reader at 490 nm.

**CD25 and CD69 staining**. 1 million of HIV-1 infected Jurkat-ZSG1 and Jurkat-NB-ZSG1 cells (day 28 post-infection) were stimulated with 1 nM of PMA. 0.1% DMSO was used as vehicle control. 24 h post-stimulation, the cells were collected and stained with CD 25 APC and CD 69 Vioblue (Miltenyi Biotect, Bergisch Gladbach, Germany) according to the manufacturer's recommendations and the stained cells were analysed by flow cytometry.

**Stimulation assays.** One million JLat 6.3 and ACH2 cells expressing NB-ZSG1 or ZSG1 and HIV-1 infected Jurkat-ZSG1 and Jurkat-NB-ZSG1 cells (day 28 post-infection) were incubated with PMA (10 nM), JQ1 (5 µM), SAHA (5 µM) or 0.1% DMSO. At 24 h post-stimulation, the supernatant samples were collected for CA ELISA assay and cells were collected for cell flow cytometry analysis.

**RNAseq.** RNA was isolated using a Qiagen RNeasy Kit (Qiagen, Hilden, Germany). One µg of total RNA was rRNA depleted using an Illumina Ribo-Zero rRNA Removal Kit, and the depletion was confirmed using an RNA 6000 Pico Chip on the Agilent Bioanalyzer. The depleted RNA was then use to create an RNAseq library using the NEBNext Ultra Directional RNA Library Prep Kit for Illumina (New England Biolabs, Ipswich, MA), and sequenced at 2 × 75 bp on an Illumina NextSeq 500. FastQ files were aligned to a composite genome including human genome (version hg38/GRCh38), HIV-1_NL4.3_ isolate (GenBank: AF324493.1) produced by the pGCH proviral plasmid ([24](#_ENREF_24)). Alignment was performed using RNA-STAR v2.3.0([54](#_ENREF_54)), using the following parameters: “--runThreadN 16 --outSAMattributes All --outFilterMultimapNmax 1”, and all other parameters at default. IGV comparison was performed on randomly sampled BAMS at the equal depth.
